# Crystal structure and Hirshfeld surface analysis of ethyl 2-[5-(3-chloro­benz­yl)-6-oxo-3-phenyl-1,6-di­hydro­pyridazin-1-yl]acetate

**DOI:** 10.1107/S2056989019007424

**Published:** 2019-05-24

**Authors:** Fouad El Kalai, Cemile Baydere, Said Daoui, Rafik Saddik, Necmi Dege, Khalid Karrouchi, Noureddine Benchat

**Affiliations:** a Laboratory of Applied Chemistry and Environment (LCAE), Faculty of Sciences, Mohamed I University, 60000 Oujda, Morocco; b Department of Physics, Faculty of Arts and Sciences, Ondokuz Mayıs University, 55139-Samsun, Turkey; c Laboratory Organic synthesis, Extraction and Valorization, Faculty of Sciences, Ain Chok, University Hassan II, Casablanca, Morocco; d Laboratory of Plant Chemistry, Organic and Bioorganic Synthesis, URAC23, Faculty of Science, BP 1014, GEOPAC Research Center, Mohammed V University, Rabat, Morocco

**Keywords:** crystal structure, pyridazine, Hirshfeld surface analysis

## Abstract

The title pyridazinone derivative, C_21_H_19_ClN_2_O_3_, is not planar. The unsubstituted phenyl ring and the pyridazine ring are inclined to each other, making a dihedral angle of 17.41 (13)° whereas the Cl-substituted phenyl ring is nearly orthogonal to the pyridazine ring [88.19 (13)°]. In the crystal, C—H⋯O hydrogen bonds generate dimers with *R*_2_^2^(10) and *R*_2_^2^(24) ring motifs which are linked by C—H⋯O inter­actions, forming chains extending parallel to the *c-*axis direction. The inter­molecular inter­actions were investigated using Hirshfeld surface analysis and two-dimensional fingerprint plots, revealing that the most significant contributions to the crystal packing are from H⋯H (44.5%), C⋯H/H⋯C (18.5%), H⋯O/H⋯O (15.6%), Cl⋯H/H⋯Cl (10.6%) and C⋯C (2.8%) contacts.

## Abstract 2

In the title pyridazinone derivative, the unsubstituted phenyl ring and the pyridazine ring are inclined to each other, making a dihedral angle of 17.41 (13)°, whereas the Cl-substituted phenyl ring is nearly orthogonal to the pyridazine ring [88.19 (13)°], C_21_H_19_ClN_2_O_3_, contains one independent mol­ecule. C—H⋯O hydrogen bonds, weak C—H⋯π and weak offset π–π stacking inter­actions stabilize the packing.

## Chemical context   

Pyridazines are an important family of six-membered aromatic heterocycles (Akhtar *et al.*, 2016 ▸). The related compound pyridazinone is an important pharmacophore with a wide range of biological applications (Asif, 2015 ▸), and its chemistry has been studied for several decades. Pyridazinones are used as scaffolds for potential drug candidates (Dubey & Bhosle, 2015 ▸; Thakur *et al.*, 2010 ▸) because of their significant potential as anti­microbial (Sönmez *et al.*, 2006 ▸), anti­depressant (Boukharsa *et al.*, 2016 ▸), anti-inflammatory (Barberot *et al.*, 2018 ▸), anti­hypertensive (Siddiqui *et al.*, 2011 ▸), analgesic (Gökçe *et al.*, 2009 ▸), anti-HIV (Livermore *et al.*, 1993 ▸), anti­convulsant (Partap *et al.*, 2018 ▸; Sharma *et al.*, 2014 ▸), cardiotonic (Wang *et al.*, 2008 ▸), anti­histaminic (Tao *et al.*, 2012 ▸), glucan synthase inhibitors (Zhou *et al.*, 2011 ▸), phospho­diesterase (PDE) inhibitors (Ochiai *et al.*, 2012 ▸) and herbicidal (Asif, 2013 ▸) agents.
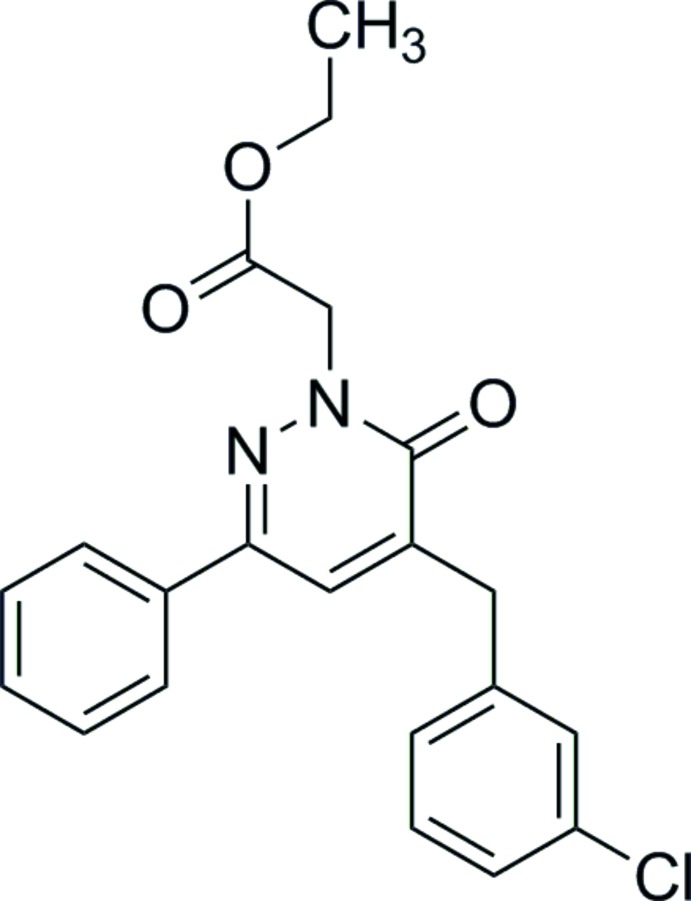


In this context and in a continuation of our work in this field (Chkirate *et al.*, 2019*a* ▸,*b* ▸; Karrouchi *et al.*, 2015 ▸, 2016*a* ▸,*b* ▸), we report herein the synthesis and the mol­ecular and crystal structures of the title pyridazinone derivative, together with its Hirshfeld surface analysis.

## Structural commentary   

**Figure 1:**
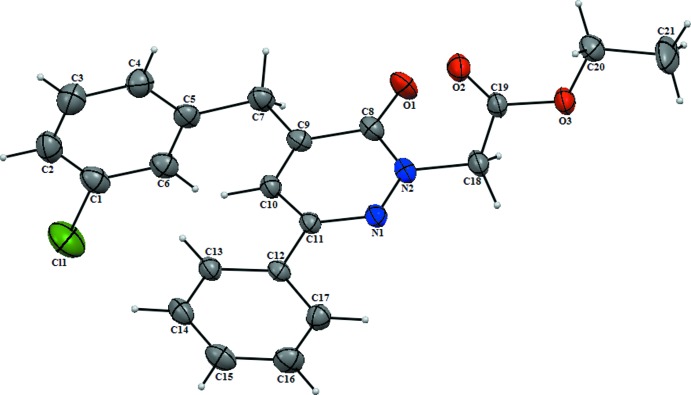
The mol­ecular structure of the title compound with displacement ellipsoids drawn at the 50% probability level.

The mol­ecule of the title compound is not planar (Fig. 1 ▸). The unsubstituted phenyl ring (C12–C17) and the pyridazine ring (C8–C11/N1/N2) are twisted relative to each other, making a dihedral angle of 17.41 (13)°; the chloro-substituted phenyl ring (C1–C6) is inclined to the pyridazine ring by 88.19 (13)°. Atoms C8 and N2 of the pyridazine ring show the largest deviations from planarity (root-mean-square deviation = 0.0236 Å) in positive and negative directions [C8 = 0.0357 (15) Å; N2 = −0.0319 (14) Å]. The O1=C8 bond length of the pyridazinone carbonyl function is 1.230 (3) Å, and the N1—N2 bond length in the pyridazine ring is 1.362 (2) Å, both in accordance with values for related pyridazinones.

## Supra­molecular features   

**Figure 2:**
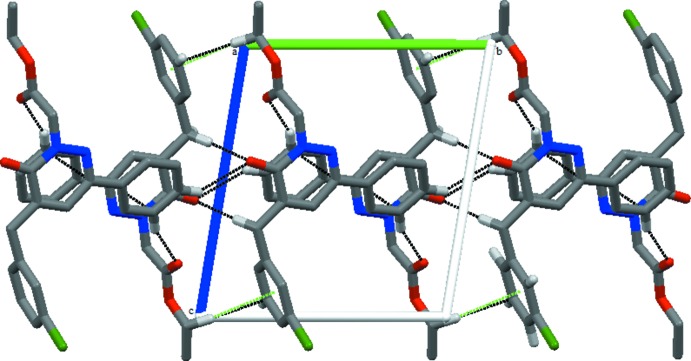
A view along the *a* axis of the crystal structure of the title compound. Black dashed lines symbolize inter­molecular C—H⋯O hydrogen bonds; C—H⋯π inter­actions are shown as green dashes lines.

The crystal packing exhibits C—H⋯O hydrogen bonds between aryl or methyl­ene groups and carbonyl O atoms (Table 1 ▸), as well as C—H⋯π inter­actions and van der Waals contacts. Inter­molecular C7—H7*B*⋯O1 and C14—H14⋯O2 hydrogen bonds produce 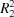
(10) and 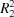
(24) motif rings (Fig. 2 ▸), supplemented by C15—H15⋯O1 contacts, forming chains extending parallel to the *c* axis (Fig. 2 ▸). A weak C20—H20*B*⋯*Cg*2 (−*x* + 1, −*y*, −*z* + 1; *Cg*2 is the centroid of the C1–C6 phenyl ring) contact is also present in this chain (Table 1 ▸; Fig. 2 ▸). Weak aromatic π–π stacking inter­actions between adjacent pyridazine rings [*Cg*1⋯*Cg*1(−*x* + 1, −*y* + 1, −*z* + 1) = 3.8833 (13) Å, where *Cg*1 is the centroid of the C8–C11/N1/N2 ring] along the *a* axis lead to the formation of a three-dimensional network.

## Database survey   

A search of the Cambridge Structural Database (CSD, version 5.40, update November 2018; Groom *et al.*, 2016 ▸) revealed two structures containing a similar pyridazinone moiety as in the title structure but with different substituents, *viz*. 4-benzyl-6-*p*-tolyl­pyridazin-3(2*H*)-one (YOTVIN; Oubair *et al.*, 2009 ▸) and ethyl 3-methyl-6-oxo-5-(3-(tri­fluoro­meth­yl)phen­yl)-1,6-di­hydro-1-pyridazine­acetate (QANVOR; Xu *et al.*, 2005 ▸). In the crystal structure of YOTVIN, the mol­ecules are connected two-by-two through N—H⋯O hydrogen bonds with an 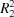
(8) graph-set motif, forming dimers arranged around an inversion center. Weak C—H⋯O hydrogen bonds and weak offset π–π stacking stabilize the packing. In the crystal structure of QANVOR, the phenyl and pyridazinone rings are approximately co-planar, making a dihedral angle of 4.84 (13)°. Centrosymmetrically related mol­ecules form dimers through non-classical inter­molecular C—H⋯O hydrogen bonds.

## Hirshfeld surface analysis   

**Figure 3:**
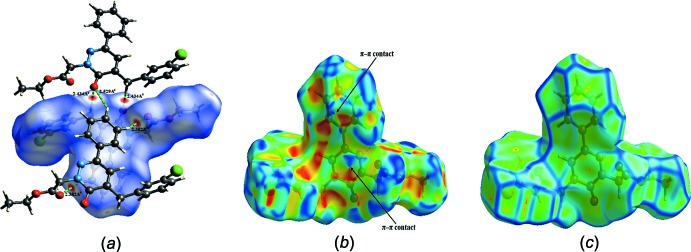
(*a*) *d*_norm_ mapped on the Hirshfeld surface for visualizing the inter­molecular inter­actions, (*b*) shape-index map of the title compound and (*c*) curvedness map of the title compound.

A Hirshfeld surface analysis (Spackman & Jayatilaka, 2009 ▸) and the associated two-dimensional fingerprint plots (McKinnon *et al.*, 2007 ▸) were performed with *CrystalExplorer17* (Turner *et al.*, 2017 ▸), using a standard (high) surface resolution with the three-dimensional *d*_norm_ surfaces plotted over a fixed colour scale of −0.1647 (red) to 1.1730 (blue) a.u. The three-dimensional *d*_norm_ surface of the title mol­ecule is illustrated in Fig. 3 ▸ *a*. The pale-red spots symbolize short contacts and negative *d*_norm_ values on the surface and correspond to the C—H⋯O inter­actions (Table 1 ▸).

The shape-index map of the title mol­ecule was generated in the range −1 to 1 Å (Fig. 3 ▸ *b*). The convex blue regions symbolize hydrogen-donor groups and the concave red regions hydrogen-acceptor groups. π–π inter­actions are generally indicated by adjacent red and blue triangles in the shape-index map, as is the case for the title mol­ecule.

The curvedness map of the title complex was generated in the range −4.0 to 0.4 Å (Fig. 3 ▸ *c*). The curvedness plot of the title complex shows large regions of green with a relatively flat (*i.e*. planar) surface area, indicating the presence of π–π stacking inter­actions, while the blue regions demonstrate areas of curvature.

**Figure 4:**
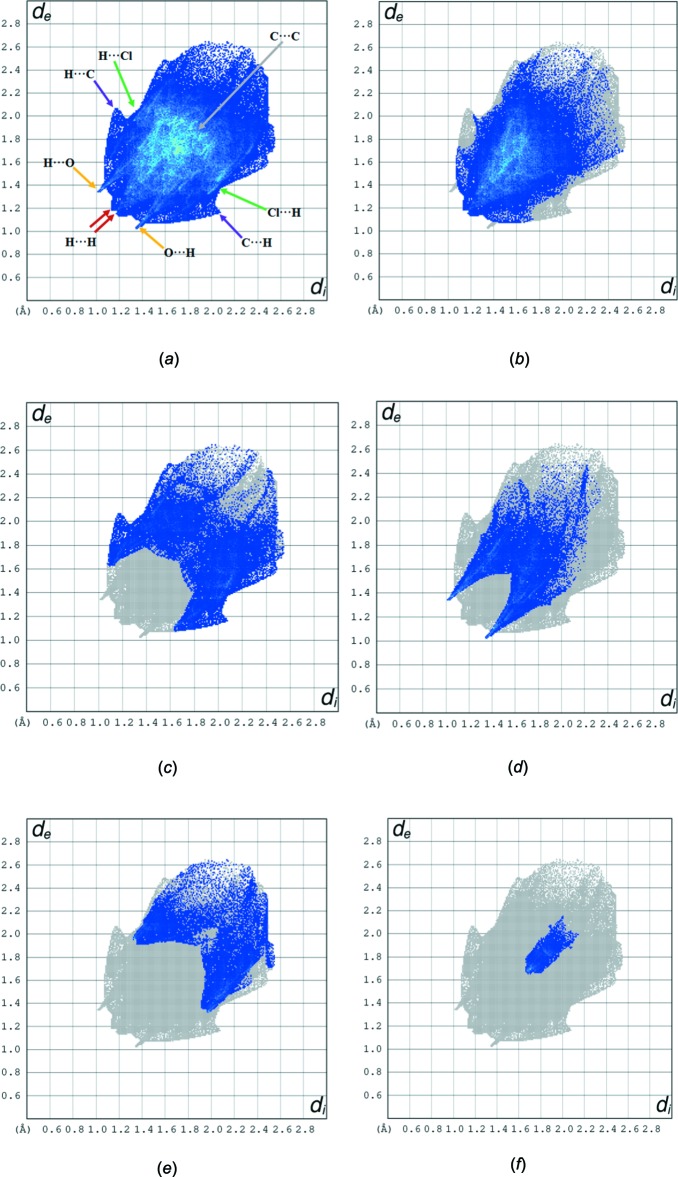
(*a*) The overall two-dimensional fingerprint plot, and delineated into (*b*) H⋯H, (*c*) H⋯C/C⋯H, (*d*) H⋯O/O⋯H, (*e*) H⋯Cl/Cl⋯H and (*f*) C⋯C inter­actions.

The overall two-dimensional fingerprint plot is illustrated in Fig. 4 ▸ *a*, delineated into H⋯H, H⋯C/ C⋯H, H⋯O/O⋯H, H⋯Cl/Cl⋯H, C⋯C contacts associated with their relative contributions to the Hirshfeld surface in Fig. 4 ▸ *b*–*f*, respectively. The most important inter­molecular inter­action is H⋯H, contributing 44.5% to the overall crystal packing, with the centre of the peak *d*_e_ = *d*_i_ = 1.18 Å (Fig. 4 ▸ *b*). H⋯C/ C⋯H contacts, with a 18.5% contribution to the Hirshfeld surface, indicate the presence of the weak C—H⋯π inter­action (Table 1 ▸). Two pairs of characteristic wings in the fingerprint plot with pairs of tips at *d*_e_ + *d*_i_ ∼2.8 Å are present (Fig. 4 ▸ *c*). H⋯O/O⋯H contacts arising from inter­molecular C—H⋯O hydrogen bonding make a 15.6% contribution to the Hirshfeld surface and are represented by a pair of sharp spikes in the region *d*_e_ + *d*_i_ ∼2.35 Å The C⋯C contacts are a measure of π–\p stacking inter­actions and contribute 2.8% of the Hirshfeld surface. They appear as an arrow-shaped distribution at *d*_e_ + *d*_i_ ∼3.3 Å. Another contact to the Hirshfeld surface is from H⋯Cl/Cl⋯H inter­actions (10.6%).

## Synthesis and crystallization   

To a solution (0.99 g, 3 mmol) of 4-(3-di­chloro­benz­yl)-6-phenyl­pyridazin-3(2*H*)-one in 30 ml of tetra­hydro­furan (THF), potassium carbonate (0.5 g, 3.5 mmol) was added. The mixture was refluxed for 1 h. After cooling, ethyl bromo­acetate (0.66 g, 4 mmol) was added and the mixture was refluxed for 8 h. The precipitated material was removed by filtration and the solvent evaporated under vacuum. The residue was purified through silica gel column chromatography using hexa­ne/ethyl acetate (4:6 *v*/*v*). Slow evaporation at room temperature led to formation of single crystals with a yield of 70%.

## Refinement   

Crystal data, data collection and structure refinement details are summarized in Table 2 ▸. Hydrogen atoms were fixed geometrically and treated as riding, with C—H = 0.97 Å for methyl [*U*_iso_(H) = 1.2*U*_eq_(C)], C—H = 0.96 Å for methyl­ene [*U*_iso_(H) = 1.5*U*_eq_(C)], C—H = 0.93 Å for aromatic [*U*_iso_(H) = 1.2*U*_eq_(C)] and C—H = 0.98 Å for methine [*U*_iso_(H) = 1.2*U*_eq_(C)] H atoms.

## Supplementary Material

Crystal structure: contains datablock(s) I. DOI: 10.1107/S2056989019007424/wm5505sup1.cif

Structure factors: contains datablock(s) I. DOI: 10.1107/S2056989019007424/wm5505Isup2.hkl

Supporting information file. DOI: 10.1107/S2056989019007424/wm5505Isup3.cml

CCDC reference: 1917654

Additional supporting information:  crystallographic information; 3D view; checkCIF report

## Figures and Tables

**Table 1:** Hydrogen-bond geometry (Å, °) *Cg*2 is the centroid of the C1–C6 phenyl ring

*D*—H⋯*A*	*D*—H	H⋯*A*	*D*⋯*A*	*D*—H⋯*A*
C14—H14⋯O2^i^	0.93	2.53	3.416 (3)	160
C7—H7*B*⋯O1^ii^	0.97	2.54	3.485 (3)	164
C15—H15⋯O1^iii^	0.93	2.66	3.474 (3)	147
C20—H20*B*⋯*Cg*2^iv^	0.97	2.81	3.759 (3)	165

Symmetry codes: (i) 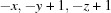; (ii) 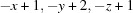; (iii) 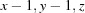; (iv) 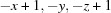.

**Table 2:** Experimental details

Crystal data	
Chemical formula	C_21_H_19_ClN_2_O_3_
*M*_r_	382.83
Crystal system, space group	Triclinic, *P* 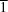
Temperature (K)	296
*a*, *b*, *c* (Å)	8.8410 (11), 10.3043 (12), 11.3610 (12)
α, β, γ (°)	94.801 (9), 103.596 (9), 106.905 (9)
*V* (Å^3^)	949.6 (2)
*Z*	2
Radiation type	Mo *K*α
μ (mm^−1^)	0.23
Crystal size (mm)	0.88 × 0.53 × 0.19

Data collection	
Diffractometer	Stoe IPDS 2
Absorption correction	Integration (*X-RED32*; Stoe & Cie, 2002 ▸)
*T*_min_, *T*_max_	0.876, 0.960
No. of measured, independent and observed [*I* > 2σ(*I*)] reflections	9612, 3716, 2058
*R*_int_	0.031
(sin θ/λ)_max_ (Å^−1^)	0.617

Refinement	
*R*[*F* ^2^ > 2σ(*F* ^2^)], *wR*(*F* ^2^), *S*	0.047, 0.127, 0.91
No. of reflections	3716
No. of parameters	245
H-atom treatment	H-atom parameters constrained
Δρ_max_, Δρ_min_ (e Å^−3^)	0.26, −0.34

Computer programs: *X-AREA* and *X-RED* (Stoe & Cie, 2002 ▸), *SHELXT2017* (Sheldrick, 2015*a* ▸), *SHELXL2018* (Sheldrick, 2015*b* ▸), *Mercury* (Macrae *et al.*, 2008 ▸), *WinGX* (Farrugia, 2012 ▸), *PLATON* (Spek, 2009 ▸) and *publCIF* (Westrip, 2010 ▸).

## supplementary crystallographic information

### Crystal data

**Table:**

C_21_H_19_ClN_2_O_3_	*Z* = 2
*M**_r_* = 382.83	*F*(000) = 400
Triclinic, *P*1	*D*_x_ = 1.339 Mg m^−^^3^
*a* = 8.8410 (11) Å	Mo *K*α radiation, λ = 0.71073 Å
*b* = 10.3043 (12) Å	Cell parameters from 11025 reflections
*c* = 11.3610 (12) Å	θ = 3.0–31.4°
α = 94.801 (9)°	µ = 0.23 mm^−^^1^
β = 103.596 (9)°	*T* = 296 K
γ = 106.905 (9)°	Prism, yellow
*V* = 949.6 (2) Å^3^	0.88 × 0.53 × 0.19 mm

### Data collection

**Table:**

Stoe IPDS 2 diffractometer	2058 reflections with *I* > 2σ(*I*)
Detector resolution: 6.67 pixels mm^-1^	*R*_int_ = 0.031
rotation method scans	θ_max_ = 26.0°, θ_min_ = 3.0°
Absorption correction: integration (X-RED32; Stoe & Cie, 2002)	*h* = −10→10
*T*_min_ = 0.876, *T*_max_ = 0.960	*k* = −12→12
9612 measured reflections	*l* = −14→14
3716 independent reflections	

### Refinement

**Table:**

Refinement on *F*^2^	0 restraints
Least-squares matrix: full	Hydrogen site location: inferred from neighbouring sites
*R*[*F*^2^ > 2σ(*F*^2^)] = 0.047	H-atom parameters constrained
*wR*(*F*^2^) = 0.127	*w* = 1/[σ^2^(*F*_o_^2^) + (0.0666*P*)^2^] where *P* = (*F*_o_^2^ + 2*F*_c_^2^)/3
*S* = 0.91	(Δ/σ)_max_ < 0.001
3716 reflections	Δρ_max_ = 0.26 e Å^−^^3^
245 parameters	Δρ_min_ = −0.34 e Å^−^^3^

### Special details

**Table:**

Geometry. All esds (except the esd in the dihedral angle between two l.s. planes) are estimated using the full covariance matrix. The cell esds are taken into account individually in the estimation of esds in distances, angles and torsion angles; correlations between esds in cell parameters are only used when they are defined by crystal symmetry. An approximate (isotropic) treatment of cell esds is used for estimating esds involving l.s. planes.

### Fractional atomic coordinates and isotropic or equivalent isotropic displacement parameters (Å^2^)

**Table:**

	*x*	*y*	*z*	*U*_iso_*/*U*_eq_	
Cl1	0.29831 (14)	0.53925 (11)	−0.10674 (8)	0.1264 (4)	
O3	0.72303 (19)	0.86361 (16)	0.93869 (13)	0.0654 (5)	
O2	0.4976 (2)	0.87323 (18)	0.80559 (15)	0.0704 (5)	
O1	0.6649 (2)	0.89797 (17)	0.57180 (15)	0.0725 (5)	
N1	0.4130 (2)	0.57611 (18)	0.60996 (15)	0.0503 (5)	
N2	0.5368 (2)	0.69655 (18)	0.62523 (15)	0.0542 (5)	
C12	0.1658 (3)	0.4089 (2)	0.49033 (17)	0.0463 (5)	
C11	0.3033 (3)	0.5381 (2)	0.50375 (17)	0.0462 (5)	
C19	0.6111 (3)	0.8315 (2)	0.83046 (19)	0.0536 (6)	
C10	0.3151 (3)	0.6166 (2)	0.40745 (18)	0.0507 (5)	
H10	0.238158	0.583782	0.331377	0.061*	
C9	0.4352 (3)	0.7377 (2)	0.42376 (19)	0.0528 (6)	
C13	0.0216 (3)	0.3784 (2)	0.39727 (19)	0.0556 (6)	
H13	0.012681	0.437926	0.340794	0.067*	
C8	0.5539 (3)	0.7869 (2)	0.5427 (2)	0.0557 (6)	
C5	0.3334 (3)	0.7688 (2)	0.2046 (2)	0.0602 (6)	
C17	0.1752 (3)	0.3177 (2)	0.5723 (2)	0.0617 (6)	
H17	0.270130	0.335520	0.636054	0.074*	
C18	0.6531 (3)	0.7357 (2)	0.7459 (2)	0.0618 (6)	
H18A	0.762208	0.780076	0.737838	0.074*	
H18B	0.654649	0.653680	0.781667	0.074*	
C14	−0.1095 (3)	0.2608 (3)	0.3867 (2)	0.0659 (7)	
H14	−0.206077	0.242734	0.324506	0.079*	
C6	0.3669 (3)	0.6883 (3)	0.1175 (2)	0.0693 (7)	
H6	0.465811	0.669538	0.134709	0.083*	
C15	−0.0967 (4)	0.1717 (3)	0.4678 (3)	0.0735 (7)	
H15	−0.183752	0.091911	0.460255	0.088*	
C7	0.4550 (3)	0.8283 (2)	0.3280 (2)	0.0659 (7)	
H7A	0.565102	0.846300	0.318471	0.079*	
H7B	0.444144	0.915570	0.356666	0.079*	
C20	0.6983 (3)	0.9534 (3)	1.0330 (2)	0.0697 (7)	
H20A	0.592464	0.912468	1.048073	0.084*	
H20B	0.702185	1.041629	1.007747	0.084*	
C16	0.0439 (4)	0.1999 (3)	0.5596 (3)	0.0749 (8)	
H16	0.051884	0.138934	0.614912	0.090*	
C1	0.2528 (4)	0.6351 (3)	0.0037 (2)	0.0752 (8)	
C4	0.1844 (4)	0.7928 (3)	0.1767 (2)	0.0752 (8)	
H4	0.160060	0.847345	0.234301	0.090*	
C2	0.1061 (4)	0.6593 (3)	−0.0218 (3)	0.0839 (9)	
H2	0.030067	0.622809	−0.097796	0.101*	
C3	0.0711 (4)	0.7371 (3)	0.0646 (3)	0.0902 (9)	
H3	−0.029726	0.752677	0.047719	0.108*	
C21	0.8330 (5)	0.9709 (4)	1.1455 (3)	0.1279 (16)	
H21A	0.936252	1.019422	1.131745	0.192*	
H21B	0.834403	0.882260	1.164831	0.192*	
H21C	0.815587	1.022374	1.212596	0.192*	

### Atomic displacement parameters (Å^2^)

**Table:**

	*U*^11^	*U*^22^	*U*^33^	*U*^12^	*U*^13^	*U*^23^
Cl1	0.1545 (9)	0.1266 (8)	0.0925 (6)	0.0370 (7)	0.0468 (6)	−0.0221 (5)
O3	0.0619 (10)	0.0768 (11)	0.0468 (8)	0.0251 (9)	−0.0023 (8)	−0.0084 (7)
O2	0.0597 (11)	0.0818 (12)	0.0620 (10)	0.0286 (9)	0.0007 (8)	−0.0056 (8)
O1	0.0687 (11)	0.0536 (10)	0.0731 (11)	−0.0058 (9)	0.0156 (9)	−0.0070 (8)
N1	0.0517 (11)	0.0514 (11)	0.0437 (10)	0.0153 (9)	0.0090 (8)	0.0008 (8)
N2	0.0504 (11)	0.0545 (11)	0.0460 (10)	0.0091 (10)	0.0048 (8)	−0.0036 (8)
C12	0.0519 (13)	0.0456 (12)	0.0411 (11)	0.0144 (10)	0.0158 (10)	0.0004 (9)
C11	0.0502 (13)	0.0478 (12)	0.0386 (11)	0.0162 (11)	0.0100 (10)	0.0005 (9)
C19	0.0475 (13)	0.0558 (13)	0.0452 (12)	0.0093 (11)	0.0012 (10)	0.0008 (9)
C10	0.0563 (14)	0.0501 (13)	0.0401 (11)	0.0141 (11)	0.0086 (10)	0.0009 (9)
C9	0.0594 (14)	0.0458 (12)	0.0505 (12)	0.0126 (11)	0.0175 (11)	0.0015 (9)
C13	0.0589 (14)	0.0537 (13)	0.0475 (12)	0.0142 (12)	0.0098 (11)	−0.0004 (10)
C8	0.0573 (15)	0.0511 (13)	0.0525 (13)	0.0119 (12)	0.0145 (11)	−0.0035 (10)
C5	0.0752 (17)	0.0482 (13)	0.0541 (13)	0.0100 (12)	0.0218 (12)	0.0158 (10)
C17	0.0624 (16)	0.0636 (15)	0.0562 (13)	0.0174 (13)	0.0130 (12)	0.0126 (11)
C18	0.0541 (14)	0.0672 (15)	0.0505 (12)	0.0165 (12)	−0.0014 (11)	−0.0082 (11)
C14	0.0514 (15)	0.0637 (16)	0.0668 (15)	0.0067 (13)	0.0084 (12)	−0.0105 (13)
C6	0.0742 (18)	0.0634 (15)	0.0690 (16)	0.0162 (13)	0.0246 (14)	0.0100 (12)
C15	0.0699 (18)	0.0566 (16)	0.0838 (18)	0.0018 (13)	0.0295 (15)	−0.0022 (14)
C7	0.0793 (18)	0.0524 (14)	0.0592 (14)	0.0114 (12)	0.0179 (13)	0.0110 (11)
C20	0.0812 (19)	0.0734 (17)	0.0524 (13)	0.0277 (14)	0.0147 (13)	−0.0022 (12)
C16	0.091 (2)	0.0592 (16)	0.0750 (17)	0.0149 (16)	0.0318 (16)	0.0214 (13)
C1	0.095 (2)	0.0646 (16)	0.0622 (16)	0.0137 (16)	0.0299 (15)	0.0051 (12)
C4	0.089 (2)	0.0779 (18)	0.0639 (16)	0.0308 (16)	0.0244 (15)	0.0151 (13)
C2	0.092 (2)	0.085 (2)	0.0601 (16)	0.0142 (17)	0.0099 (16)	0.0108 (14)
C3	0.088 (2)	0.101 (2)	0.083 (2)	0.0356 (18)	0.0183 (17)	0.0193 (17)
C21	0.156 (3)	0.171 (4)	0.0489 (16)	0.091 (3)	−0.0186 (19)	−0.0337 (18)

### Geometric parameters (Å, º)

**Table:**

Cl1—C1	1.724 (3)	C17—H17	0.9300
O3—C19	1.331 (2)	C18—H18A	0.9700
O3—C20	1.454 (3)	C18—H18B	0.9700
O2—C19	1.187 (3)	C14—C15	1.362 (4)
O1—C8	1.230 (3)	C14—H14	0.9300
N1—C11	1.304 (2)	C6—C1	1.391 (4)
N1—N2	1.362 (2)	C6—H6	0.9300
N2—C8	1.378 (3)	C15—C16	1.359 (4)
N2—C18	1.450 (3)	C15—H15	0.9300
C12—C17	1.383 (3)	C7—H7A	0.9700
C12—C13	1.385 (3)	C7—H7B	0.9700
C12—C11	1.487 (3)	C20—C21	1.484 (4)
C11—C10	1.420 (3)	C20—H20A	0.9700
C19—C18	1.502 (3)	C20—H20B	0.9700
C10—C9	1.347 (3)	C16—H16	0.9300
C10—H10	0.9300	C1—C2	1.360 (4)
C9—C8	1.447 (3)	C4—C3	1.378 (4)
C9—C7	1.500 (3)	C4—H4	0.9300
C13—C14	1.386 (3)	C2—C3	1.363 (4)
C13—H13	0.9300	C2—H2	0.9300
C5—C6	1.378 (3)	C3—H3	0.9300
C5—C4	1.380 (4)	C21—H21A	0.9600
C5—C7	1.503 (3)	C21—H21B	0.9600
C17—C16	1.384 (4)	C21—H21C	0.9600
			
C19—O3—C20	116.13 (18)	C13—C14—H14	120.1
C11—N1—N2	116.83 (18)	C5—C6—C1	120.0 (3)
N1—N2—C8	126.86 (17)	C5—C6—H6	120.0
N1—N2—C18	114.58 (19)	C1—C6—H6	120.0
C8—N2—C18	118.35 (19)	C16—C15—C14	119.7 (2)
C17—C12—C13	117.8 (2)	C16—C15—H15	120.1
C17—C12—C11	121.31 (19)	C14—C15—H15	120.1
C13—C12—C11	120.81 (19)	C9—C7—C5	114.18 (19)
N1—C11—C10	121.6 (2)	C9—C7—H7A	108.7
N1—C11—C12	116.04 (18)	C5—C7—H7A	108.7
C10—C11—C12	122.40 (17)	C9—C7—H7B	108.7
O2—C19—O3	125.0 (2)	C5—C7—H7B	108.7
O2—C19—C18	126.11 (19)	H7A—C7—H7B	107.6
O3—C19—C18	108.9 (2)	O3—C20—C21	106.6 (2)
C9—C10—C11	121.50 (19)	O3—C20—H20A	110.4
C9—C10—H10	119.3	C21—C20—H20A	110.4
C11—C10—H10	119.3	O3—C20—H20B	110.4
C10—C9—C8	118.4 (2)	C21—C20—H20B	110.4
C10—C9—C7	125.0 (2)	H20A—C20—H20B	108.6
C8—C9—C7	116.5 (2)	C15—C16—C17	121.1 (3)
C12—C13—C14	121.3 (2)	C15—C16—H16	119.4
C12—C13—H13	119.4	C17—C16—H16	119.4
C14—C13—H13	119.4	C2—C1—C6	120.6 (3)
O1—C8—N2	120.3 (2)	C2—C1—Cl1	119.6 (2)
O1—C8—C9	125.2 (2)	C6—C1—Cl1	119.8 (3)
N2—C8—C9	114.5 (2)	C3—C4—C5	120.9 (3)
C6—C5—C4	118.5 (2)	C3—C4—H4	119.6
C6—C5—C7	121.1 (3)	C5—C4—H4	119.6
C4—C5—C7	120.4 (2)	C1—C2—C3	119.7 (3)
C12—C17—C16	120.2 (2)	C1—C2—H2	120.1
C12—C17—H17	119.9	C3—C2—H2	120.1
C16—C17—H17	119.9	C2—C3—C4	120.3 (3)
N2—C18—C19	112.24 (19)	C2—C3—H3	119.9
N2—C18—H18A	109.2	C4—C3—H3	119.9
C19—C18—H18A	109.2	C20—C21—H21A	109.5
N2—C18—H18B	109.2	C20—C21—H21B	109.5
C19—C18—H18B	109.2	H21A—C21—H21B	109.5
H18A—C18—H18B	107.9	C20—C21—H21C	109.5
C15—C14—C13	119.8 (2)	H21A—C21—H21C	109.5
C15—C14—H14	120.1	H21B—C21—H21C	109.5

### Hydrogen-bond geometry (Å, º)

*Cg*2 is the centroid of the C1–C6 phenyl ring

**Table:**

*D*—H···*A*	*D*—H	H···*A*	*D*···*A*	*D*—H···*A*
C14—H14···O2^i^	0.93	2.53	3.416 (3)	160
C7—H7*B*···O1^ii^	0.97	2.54	3.485 (3)	164
C15—H15···O1^iii^	0.93	2.66	3.474 (3)	147
C20—H20*B*···*Cg*2^iv^	0.97	2.81	3.759 (3)	165

Symmetry codes: (i) −*x*, −*y*+1, −*z*+1; (ii) −*x*+1, −*y*+2, −*z*+1; (iii) *x*−1, *y*−1, *z*; (iv) −*x*+1, −*y*, −*z*+1.
